# Supplementary material for: Learning functional groups in complex microbiomes
Source: bioRxiv. 2026 Mar 3:2026.03.03.709366. Preprint. [Version 1] doi: 10.64898/2026.03.03.709366 (PMC13001407; doi:10.64898/2026.03.03.709366)
Supplement: Supplement 1 [file NIHPP2026.03.03.709366v1-supplement-1.pdf]

bioRxiv preprint doi: https://doi.org/10.64898/2026.03.03.709366; this version posted March 3, 2026. The copyright holder for this preprint (which was not certified by peer review) is the author/funder, who has granted bioRxiv a license to display the preprint in perpetuity. It is made available under aCC-BY 4.0 International license.

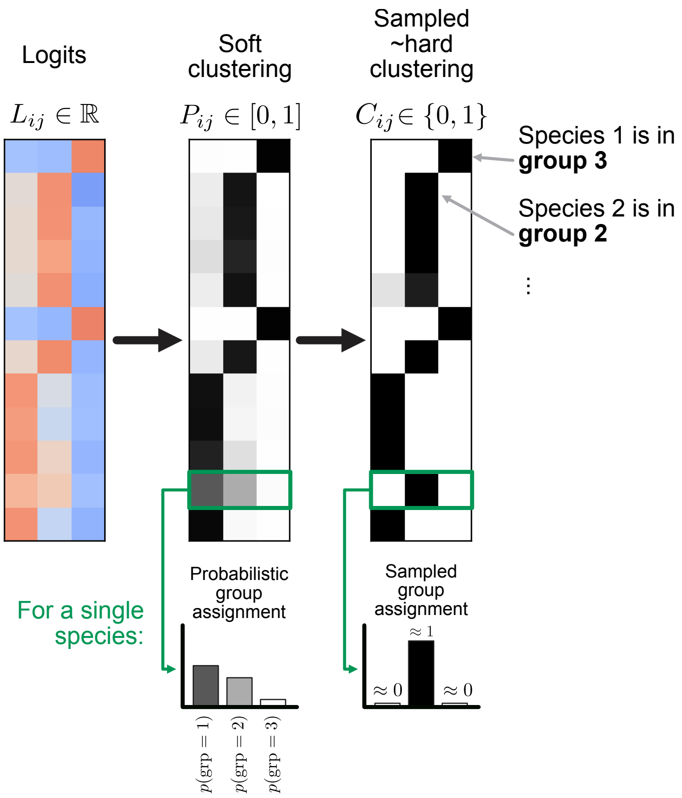

Supplementary Figure S1: Illustration of the Gumbel softmax trick for generating a clustering matrix The central matrix P denotes the probabilistic clustering matrix. It is derived (via softmax) from the matrix L to the left, the real-valued matrix of logits which are updated by gradient descent. Each row of P describes the probabilistic group assignment of a single species to different groups. From P, an approximately *hard* clustering matrix C is sampled using the Gumbel softmax trick described in Methods. Each row is approximately a one-hot vector that describes the group identity of the species.

bioRxiv preprint doi: https://doi.org/10.64898/2026.03.03.709366; this version posted March 3, 2026. The copyright holder for this preprint (which was not certified by peer review) is the author/funder, who has granted bioRxiv a license to display the preprint in perpetuity. It is made available under aCC-BY 4.0 International license.

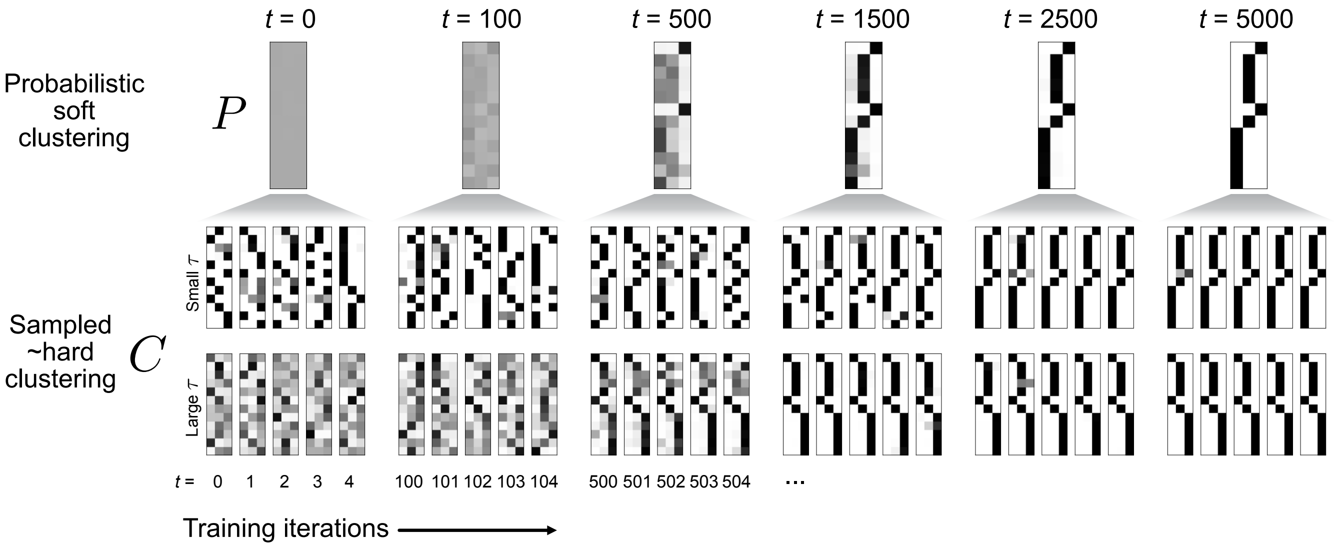

Supplementary Figure S2: Illustration of P and C matrices during training on a toy dataset. (Top) The probabilistic clustering P evolves slowly during training. (Bottom two rows) At each training step, an approximately hard clustering matrix C is sampled. For small τ (middle) this approximation is closer.

bioRxiv preprint doi: https://doi.org/10.64898/2026.03.03.709366; this version posted March 3, 2026. The copyright holder for this preprint (which was not certified by peer review) is the author/funder, who has granted bioRxiv a license to display the preprint in perpetuity. It is made available under aCC-BY 4.0 International license.

SUPPLEMENTARY INFORMATION

bioRxiv preprint doi: https://doi.org/10.64898/2026.03.03.709366; this version posted March 3, 2026. The copyright holder for this preprint (which was not certified by peer review) is the author/funder, who has granted bioRxiv a license to display the preprint in perpetuity. It is made available under aCC-BY 4.0 International license.

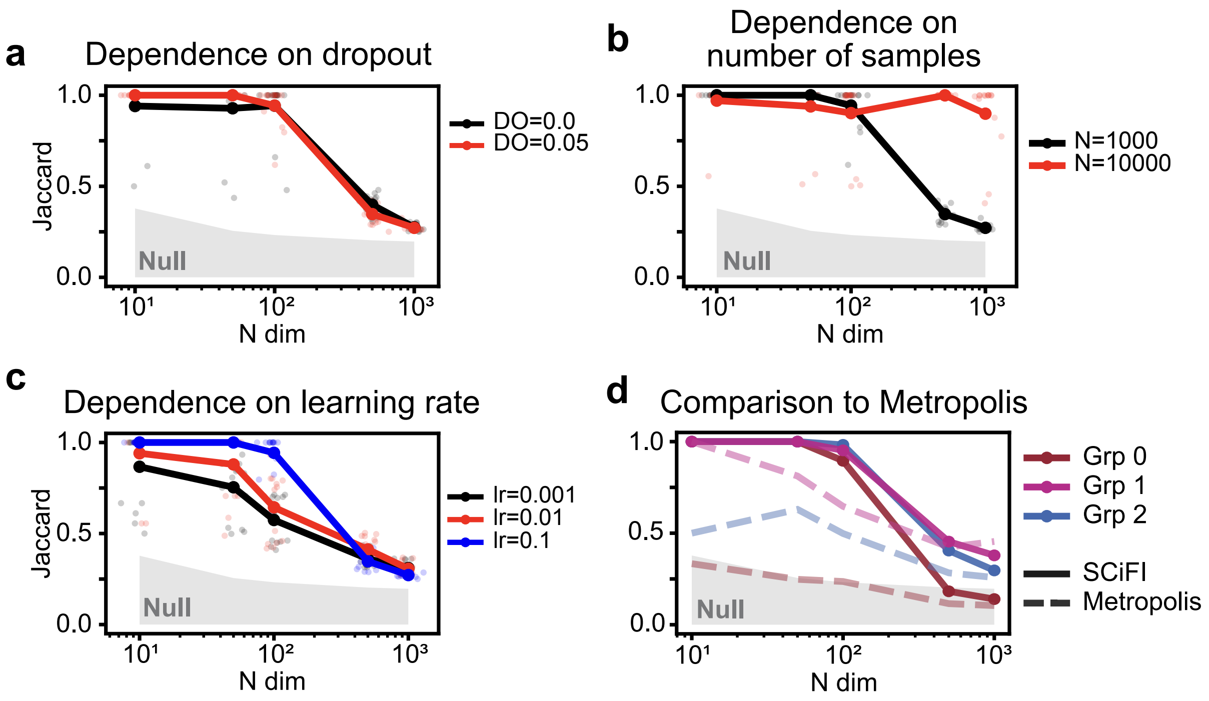

Supplementary Figure S3: Training hyperparameters impacting performance on synthetic data. We explore how SCiFI's ability to recover the correct groups depends on critical training parameters and on the dimension of the input. The synthetic data follows the structure discussed in Methods and used in Fig. 2. The basic group structure is $\hat{c} = (1, 2, 2, 2, 3, 3, 3, 3, 3, 3)$ which assigns 10 species to 3 clusters. We vary the dimensionality of the system while keeping the group number fixed by simply repeating $\hat{c}$ for an integer number of repetitions (e.g. 10 repetitions would correspond to a dimensionality of 100). The neural network used to model the structure function map is small, consisting of 2 layers of 48 neurons each. This small network is sufficient because the function is piecewise linear. In all cases we measure group recovery with the Jaccard index (see Methods). (a) Variation of group recovery as a function of dimensionality for different values of dropout. Every faint dot corresponds to one model of an ensemble ($N_{\text{ens}} = 15$), slightly jiggled in the x-direction for visualization. The solid dots with connecting line are the average Jaccard index across all ensemble members. The number of samples used for training is 1000 and we see performance start to decrease at $N_{\text{dim}} = 100$. Dropout makes negligible difference to the group recovery, but it significantly improves generalization (not shown). (b) Same as in (a) but varying the number of training samples. Performance increases significantly when the number of samples is increased. This suggests that given enough data, SCiFI should find the correct groups. (c) Same as in (c) but varying the learning rate during training. In general, learning rate can significantly impact performance. Here we see that using a larger learning rate improves group recovery. However, too large and the networks will fail to train (not shown). (d) Comparison of SCiFI group recovery to the Metropolis Hastings algorithm from Ref. [25]. Here we show the recovery of each group individually. SCiFI generically outperforms the Metropolis Hastings algorithm. Here we also see that the group recovery differs across groups. This suggests that the decrease in performance at $N_{\text{dim}} \approx 100$ is due to the particular structure-function map used.

bioRxiv preprint doi: https://doi.org/10.64898/2026.03.03.709366; this version posted March 3, 2026. The copyright holder for this preprint (which was not certified by peer review) is the author/funder, who has granted bioRxiv a license to display the preprint in perpetuity. It is made available under aCC-BY 4.0 International license.

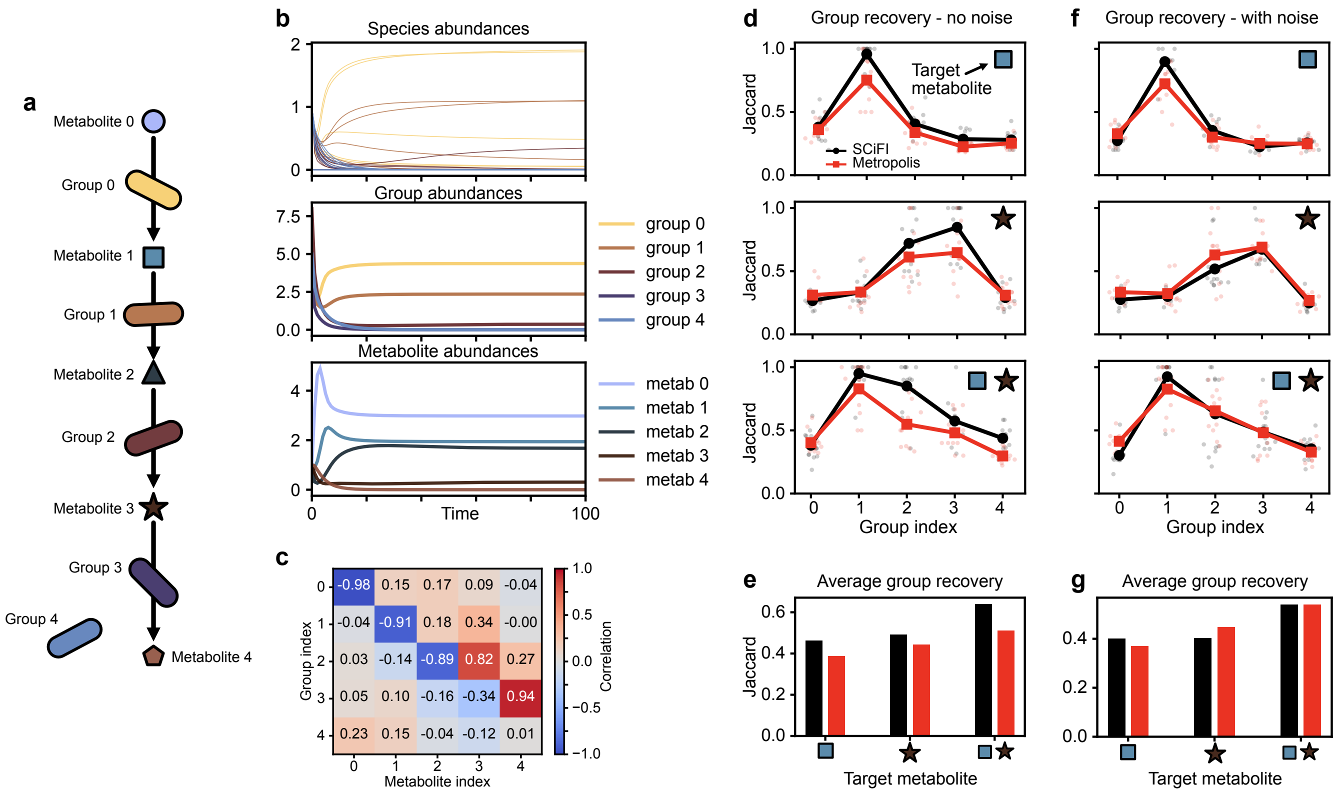

Supplementary Figure S4: SCiFI applied to a linear degradation chain. (a) Here we evaluate SCiFI on a simulated community of species which participate in a linear degradation chain of a metabolite as in Ref. [25]. The first four groups participate in the chain, the last group does not. (b) Evolution in time of the species abundances (top), grouped abundances (middle) and metabolite abundances (bottom). (c) Correlations between end-point group abundances and metabolite abundances. (d) Group recovery as measured by the Jaccard index for each group. Red shows the results of the Metropolis-Hastings algorithm from Ref. [25], black shows SCiFI. The icon in the upper right indicates what function (metabolite abundance) was used to train SCiFI. The top row uses metabolite 1, middle uses metabolite 3, and bottom uses both. (e) Average group recovery across all groups, separated by target metabolite algorithm (SCiFI - black, Metropolis - red) . Group recovery improves when using multiple intermediate metabolites (right columns). (f-g) Same as in (d-e) but with added 10% noise on the metabolites concentrations. We see that the performance gap between SCiFI and Metropolis decreases. This is unsurprising because Ref. [25] shows that the Metropolis algorithm works well in this setting.

bioRxiv preprint doi: https://doi.org/10.64898/2026.03.03.709366; this version posted March 3, 2026. The copyright holder for this preprint (which was not certified by peer review) is the author/funder, who has granted bioRxiv a license to display the preprint in perpetuity. It is made available under aCC-BY 4.0 International license.

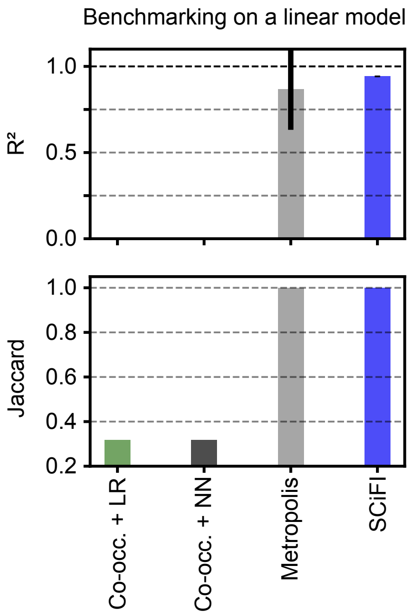

Supplementary Figure S5: Benchmarking on a linear model. This plot compares the same models as in Fig. 2d-e, but when the true structure-function map is linear. In this case we see that Model III (the Metropolis-Hastings algorithm from Ref. [25]) recovers the groups correctly. The co-occurrence models perform poorly because in this simulation there is no built-in correlation structure among different species.

bioRxiv preprint doi: https://doi.org/10.64898/2026.03.03.709366; this version posted March 3, 2026. The copyright holder for this preprint (which was not certified by peer review) is the author/funder, who has granted bioRxiv a license to display the preprint in perpetuity. It is made available under aCC-BY 4.0 International license.

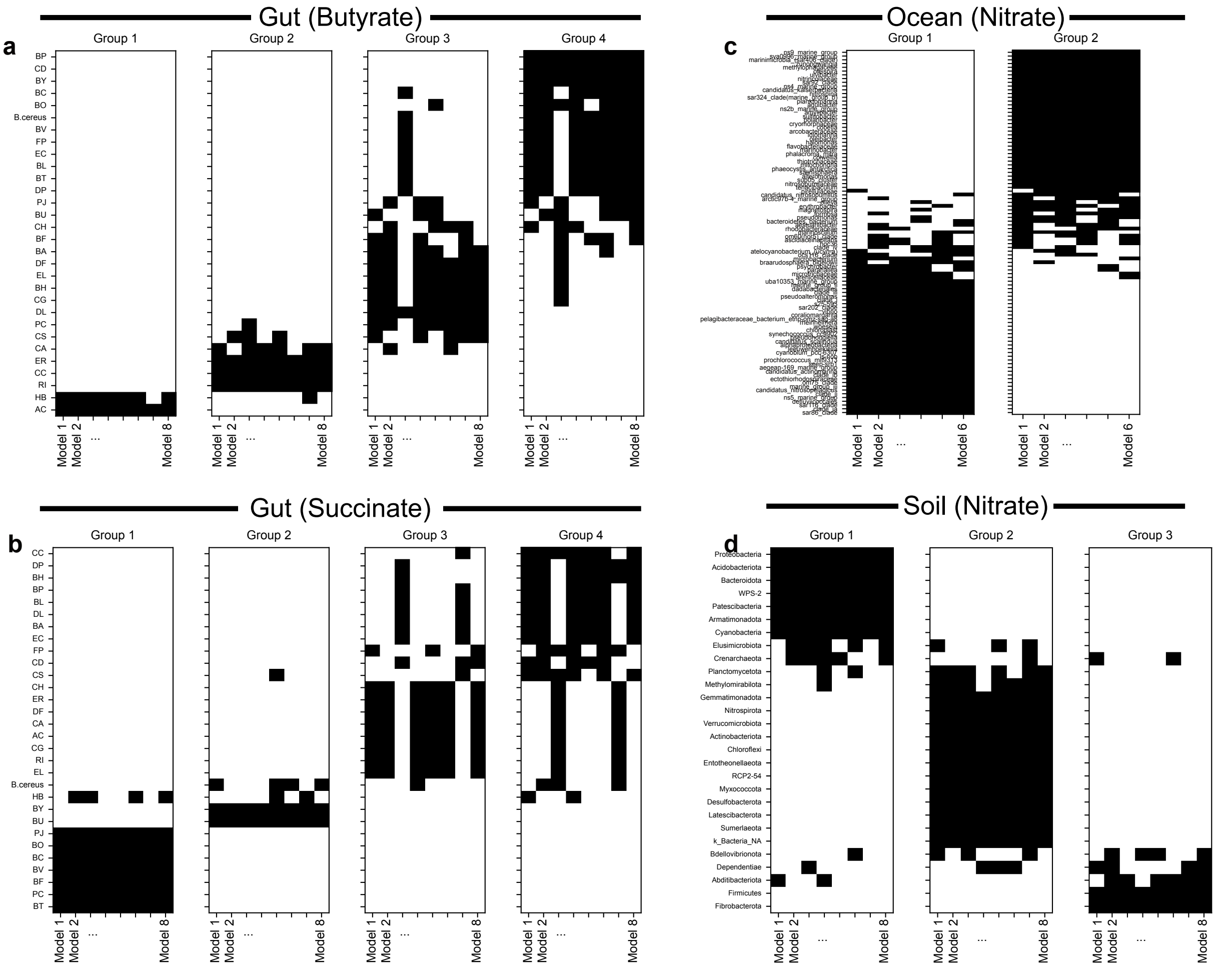

Supplementary Figure S6: Consistency of groups across test-train splits. For each of the datasets in Fig. 3 we illustrate the consistency of the learned groups across the different test/train splits used for cross validation. As a reminder, we train an ensemble of models with different test train splits (Methods). (a) Each matrix (left to right) corresponds to one group. Each column of the matrices in this figure corresponds to one model. Each row corresponds to one species. Black means the species is included in the corresponding group, white means it is not. For example, AC is always in Group 1, and CC is always in Group 2. Because the indices of the groups are arbitrary, we must reorder them so they are consistent across models. We do this by ordering the groups by their correlation with the target function. This process is imperfect as can be seen in Group 3 and Group 4, where it is clear that the assignments of the groups have been switched but the composition is unchanged. (b) Corresponding plots for the gut data when trained to predict succinate using four groups (note that only two groups are used in Fig. 3g-h). (c) Corresponding plots for the ocean data when predicting nitrate. (c) Corresponding plots for the soil data when predicting nitrate dynamics.

bioRxiv preprint doi: https://doi.org/10.64898/2026.03.03.709366; this version posted March 3, 2026. The copyright holder for this preprint (which was not certified by peer review) is the author/funder, who has granted bioRxiv a license to display the preprint in perpetuity. It is made available under aCC-BY 4.0 International license.

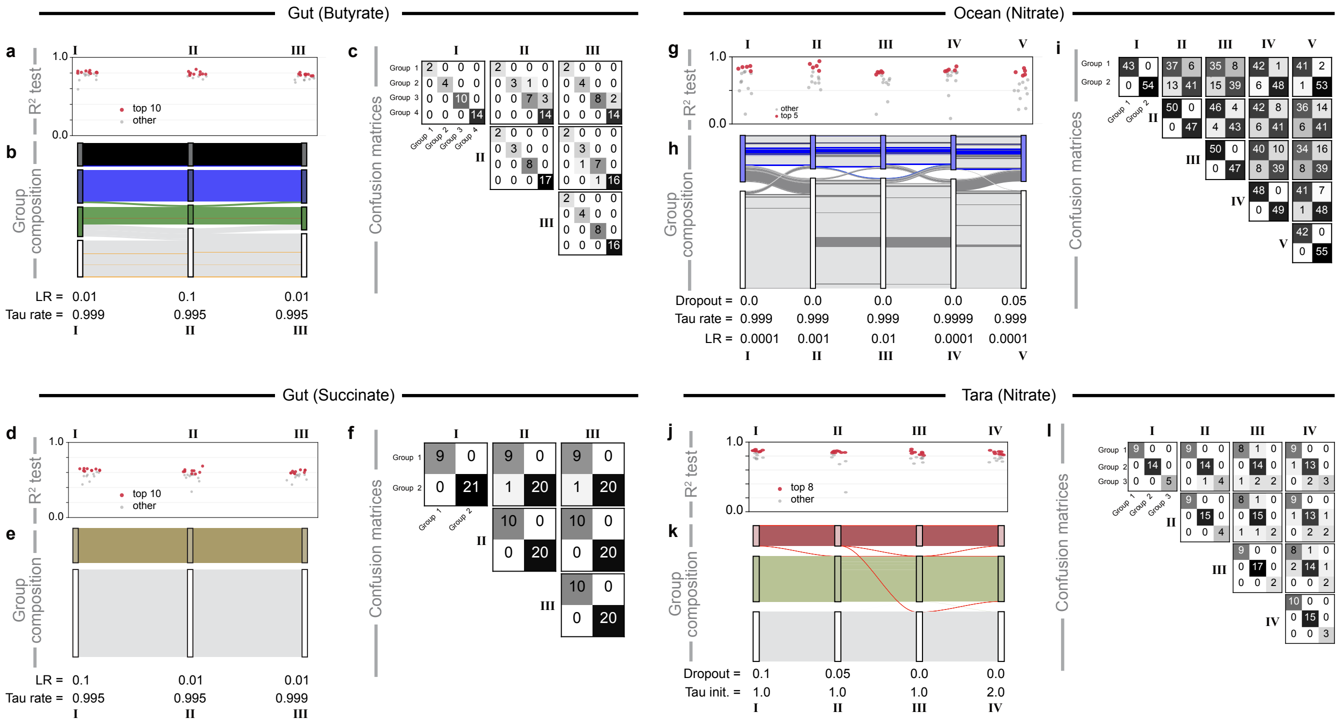

Supplementary Figure S7: Consistency of groups across hyperparameters. For each of the datasets in Fig. 3 we evaluate the sensitivity of the learned groups to hyperparameters used for model training (Methods). For each of these datasets we show three plots which follow the same structure. (a) R^2 values for each of the different hyperparameter sets, enumerated with Roman numerals. Small dots correspond to single models in the ensemble. The top models highlighted in red are used to determine the consensus grouping (Methods). (b) Consensus group composition resulting for each hyperparameter set. Each set of nodes (rectangles) in the flow diagram are aligned vertically with the corresponding hyperparameter set from (a). Each bar between nodes represents one species; its thickness corresponds to its average abundance. While the grouping is very stable for the synthetic gut data, it is less so for the ocean data (panels g-i). The hyperparameters corresponding to each set (I-III) are shown at the bottom of (b). (c) Confusion matrices to compare the group assignments across hyperparameter sets. Each matrix is a comparison between one set of hyperparameters (Roman numerals). Entry i, j in the matrix is the number of species that are assigned to group i for one hyperparameter set and to group j for another hyperparameter set. (d-f) Corresponding plots for the gut data with succinate concentration as the target function. (g-i) Corresponding plots for Tara oceans data with nitrate concentration as the target function. Blue species which were identified by the EQO method from Ref. [24] are highlighted in blue. In this data (as opposed to the synthetic gut data) there is more variation in the relevant (top) group which reflects the fact that the data is both more complex (higher dimensionality) and less sampled. Species colored in dark gray are ones that are sometimes, but not always, in the relevant group. (j-l) Corresponding plots for the soil data with nitrate dynamics as the target function.

bioRxiv preprint doi: https://doi.org/10.64898/2026.03.03.709366; this version posted March 3, 2026. The copyright holder for this preprint (which was not certified by peer review) is the author/funder, who has granted bioRxiv a license to display the preprint in perpetuity. It is made available under aCC-BY 4.0 International license.

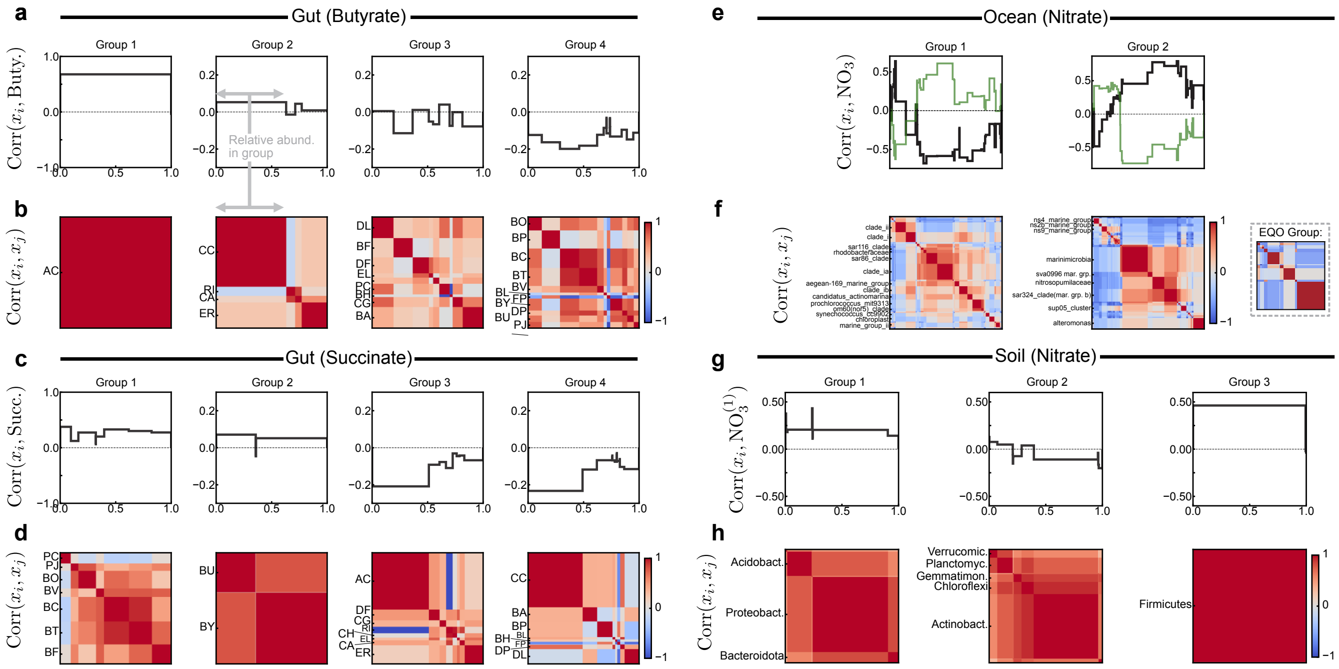

Supplementary Figure S8: Species-species and species-function correlations. Here we investigate the composition of the groups in each of the datasets in Fig. 3. For each dataset we visualize the correlations between individual species and the target function (top row, line plot) and species-species correlations (bottom row, image plot). (a) For each group (column) we plot the correlation of the constituent species with butyrate. The order of the species is the same as in (b) and they are aligned visually. The width of each species corresponds to its relative abundance in the group. Each block in the images in i, j in (b) show the correlation between species i and species j (red is positive, blue is negative). For example, AC completely dominates Group 1 and has a correlation of ∼0.7 with butyrate. Group 2 is composed of four species. CC and CA are slightly positively correlated with butyrate, while ER is not correlated (despite being a direct producer). CC has the largest relative abundance in the group by far. (c-d) Same plots for the synthetic gut data when succinate is the target function. We observe two groups that are positively correlated with succinate (Group 1 and 2), while the others are negatively correlated. (e-f) Plots for the ocean data in Fig. 3i-l. Group 2 is the group that contains all species identified by the EQO method from Ref. [24]. (e) In addition to showing how each species is correlated with nitrate (black) we show how they are correlated with oxygen (green). Typically these correlations are opposite sign, except for several species in Group 2. This suggests a similar group composition as identified by EQO [24] (shown to the right), where species with different, "complementary" strategies are grouped to form a functional group that is informative across all environmental conditions. In this case, high and low oxygen concentration. (g-h) Plots for the soil data in Fig. 3m-p.

bioRxiv preprint doi: https://doi.org/10.64898/2026.03.03.709366; this version posted March 3, 2026. The copyright holder for this preprint (which was not certified by peer review) is the author/funder, who has granted bioRxiv a license to display the preprint in perpetuity. It is made available under aCC-BY 4.0 International license.

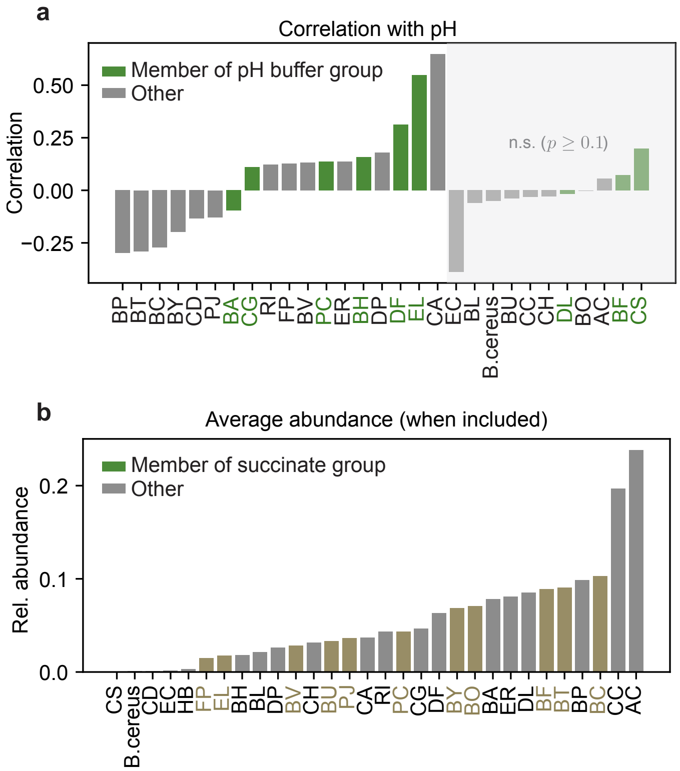

Supplementary Figure S9: Gut species abundances and correlations with pH. (a) Correlation of every species with pH in samples where that species was included. Samples with p value greater than 10^{-1} are on the right. Species in the "pH buffer group" are highlighted in green. (b) Average abundance of species across the dataset (not stratified by community richness). Species in the relevant succinate group are highlighted in brown.

bioRxiv preprint doi: https://doi.org/10.64898/2026.03.03.709366; this version posted March 3, 2026. The copyright holder for this preprint (which was not certified by peer review) is the author/funder, who has granted bioRxiv a license to display the preprint in perpetuity. It is made available under aCC-BY 4.0 International license.

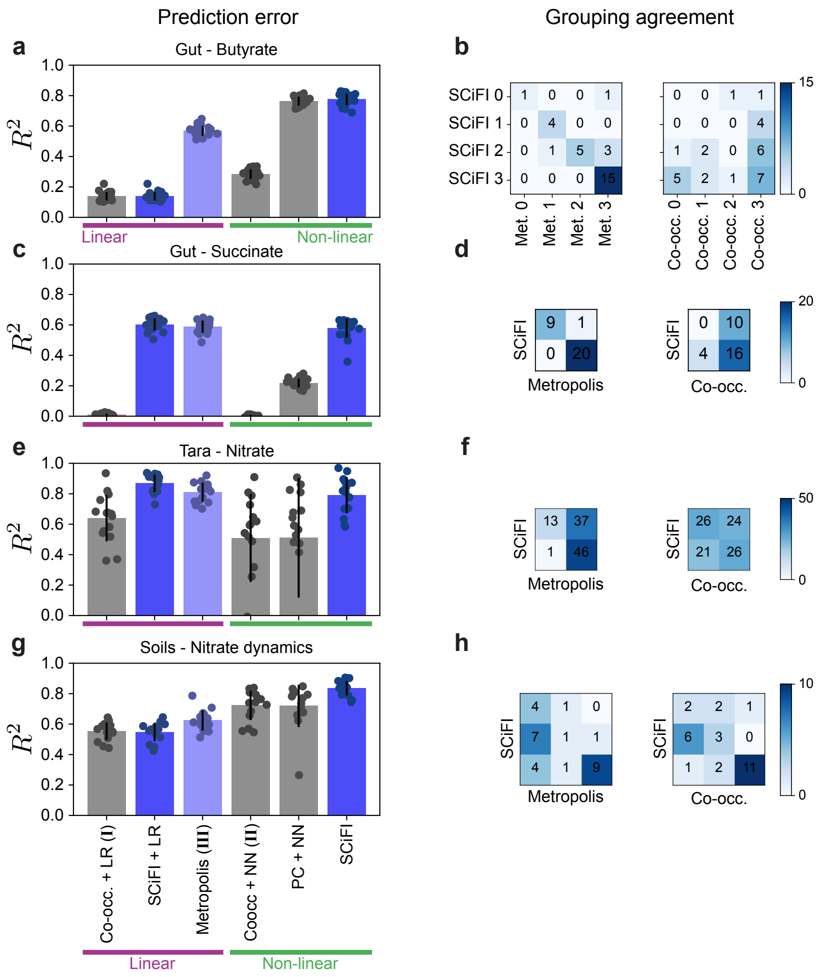

Supplementary Figure S10: Method comparison. Each row corresponds to one row (dataset) in Fig. 3. We compare SCiFI to five other approaches. For the clustering step, we consider either a co-occurrence network approach (Co-occ.), the clustering learned by SCiFI, or a linear function-informed clustering method that relies on a Metropolis-Hastings algorithm [25]. The co-occurrence clusters are either used to train a linear regression model (LR), corresponding to a Model I-type algorithm discussed the main text and Fig. 2, or to train a neural network (NN), corresponding to a Model II-type algorithm. In addition, we also consider a model which performs dimensionality reduction with principal component analysis (PCA). Left shows the R^2 of these six different models. The first three have linear structure-function maps, the last three are nonlinear. The bars show the average R^2 across the entire ensemble of models (N_{ens} =12-20 depending on dataset), with individual points showing the R^2 for single models. Blue bars are function-informed. Dark blue denotes the two approaches that rely on SCiFI: either the full SCiFI algorithm (far right), or an approach which fully trains SCiFI but then discards the neural network structure-function map for a linear model. Light blue denotes the Metropolis-Hastings algorithm which is function-informed but linear. (a) For butyrate prediction in the gut community experiments of Ref. [32], SCiFI outperforms all other models except one which uses PCA and a neural network. This indicates nonlinearity is important, and that the dominant modes of covariance in abundance data are reflective of butyrate producers. (b) Group overlap (confusion matrices) between SCiFI and the Metropolis-Hastings algorithm (left) and between SCiFI and the co-occurrence network (right). Entry i, j in the matrix shows the number of species that were assigned to group i using one method and j using the other method. In this dataset, Metropolis Hastings recovers almost the same groups as SCiFI despite not making accurate predictions. (c-d) Same as (a-b) but for succinate prediction. (e-f) Same for the Tara Oceans dataset predicting nitrate. (g-h) Same for the prediction of nitrate reduction dynamics in soils. SCiFI is the only method that either matches or outperforms all other methods across every dataset.

bioRxiv preprint doi: https://doi.org/10.64898/2026.03.03.709366; this version posted March 3, 2026. The copyright holder for this preprint (which was not certified by peer review) is the author/funder, who has granted bioRxiv a license to display the preprint in perpetuity. It is made available under aCC-BY 4.0 International license.

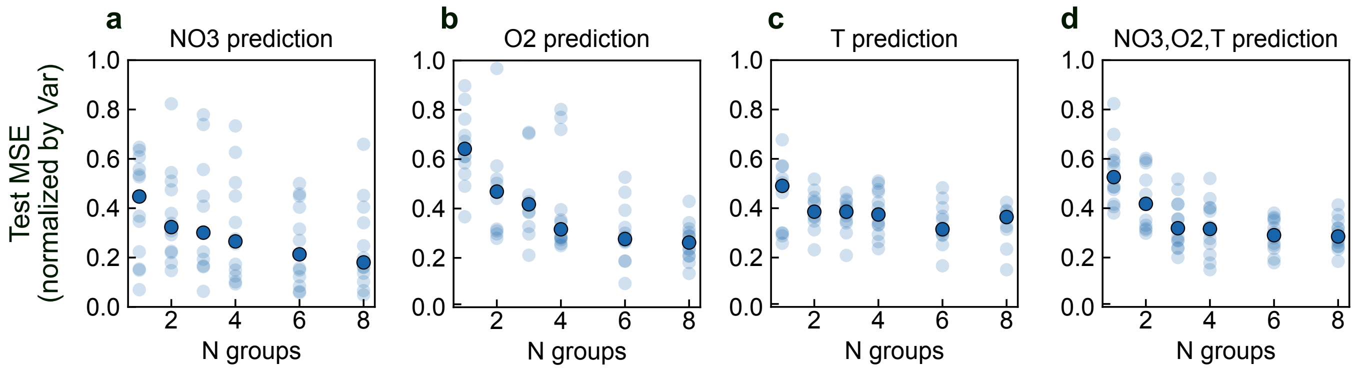

Supplementary Figure S11: Error dependence on number of groups for Tara Oceans metagenome Each panel shows the evolution of mean squared error (MSE) on a held-out test set for varying numbers of groups. MSE is normalized by the variance of the predicted function, so that a value of 1 corresponds to the error of a model which simply predicts the mean function value. As in Fig. 3, each faint dot corresponds to one model trained on one subset of the training data. Solid dots are the median over the ensemble of models. (a) Results when training SCiFI to predict nitrate concentration. Although the median decreases steadily with increasing numbers of groups, the variance is very large. (b) Results when predicting oxygen concentration. As with nitrate, the variance of model performance is large. (c) Results when predicting temperature. (d) Results when simultaneously predicting nitrate, oxygen, and temperature.

bioRxiv preprint doi: https://doi.org/10.64898/2026.03.03.709366; this version posted March 3, 2026. The copyright holder for this preprint (which was not certified by peer review) is the author/funder, who has granted bioRxiv a license to display the preprint in perpetuity. It is made available under aCC-BY 4.0 International license.

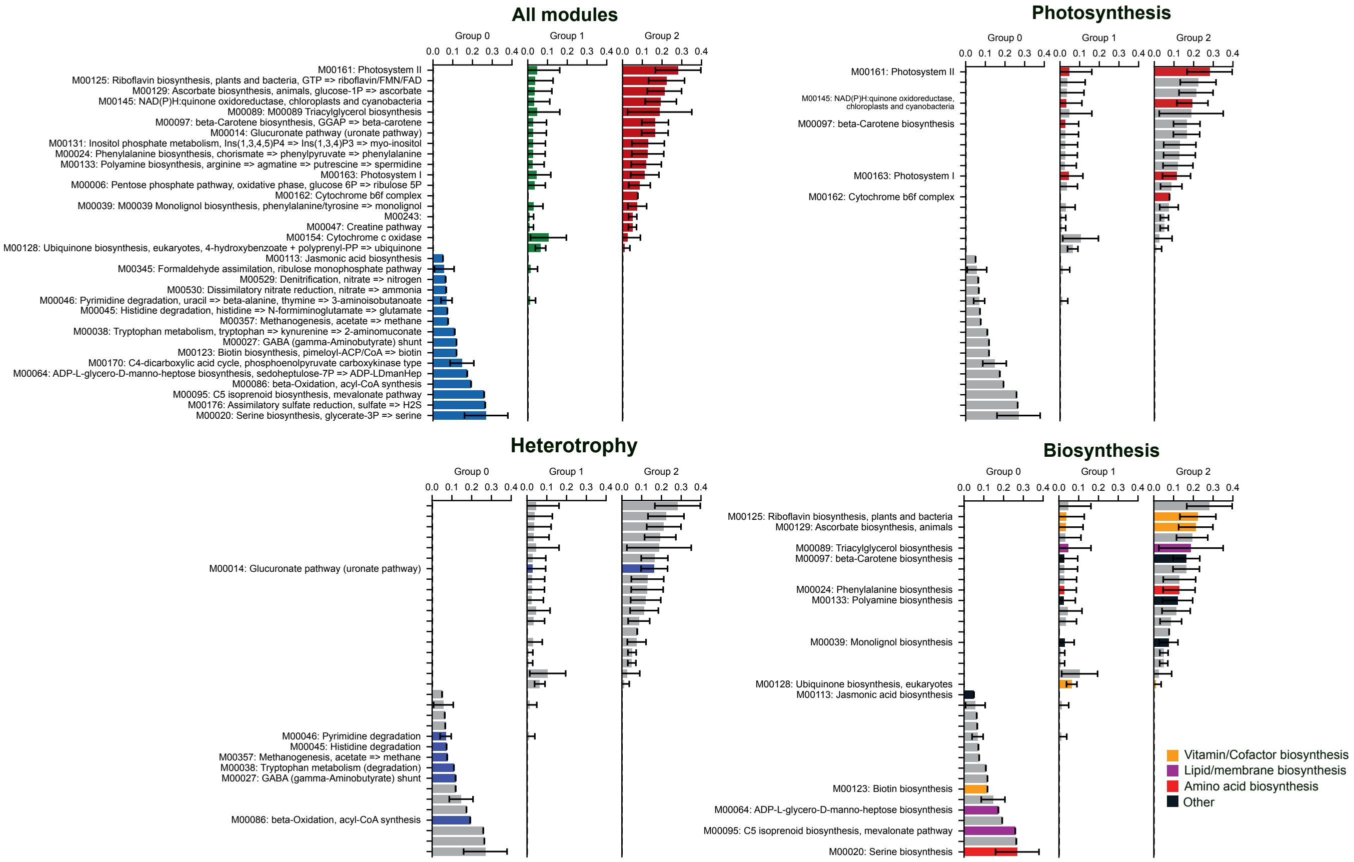

Supplementary Figure S12: Module-level cluster assignments for Tara metagenomics. Here we highlight individual modules isolated using function-driven clustering. Top left shows all KEGG modules with their full descriptions. The bar length is the average abundance of the module assigned to each cluster. This average is taken over samples, and over an ensemble of models (we compute the average only over the top 50% of models). Error bars show the standard deviation (truncated at 0) across the ensemble of models. Top right shows how modules related to photosynthesis are concentrated in group 2. Bottom left shows modules related to heterotrophy, which are mostly concentrated in group 0 with the exception of M00014 (Glucuronate pathway). Glucuronate is a precursor for building of extracellular polymeric substances (in bacteria) [39, 40]. It may also serve as a precursor to ascorbate in animals, suggesting that this may signal the presence of genetic material from eukaryotic plankton which use the protective effects of ascorbate [55]. Bottom right shows modules related to biosynthesis. These are distributed across groups 1 and 3. The surface group (group 3) contains modules used for protective structures (ascorbate, beta carotene, polyamines) and energetically-expensive structures (phenylalanine, monolignol). Riboflavin biosynthesis is important in the surface group because riboflavin degrades rapidly due to light [56], meaning it must be constantly generated to maintain the constant supply necessary for growth. The deep group (group 1) contains structures used in archea (Mevalonate, ADP-heptose), simple amino acids (serine) and cofactors which are potentially useful for hetertrophy (biotin, used for anaplerosis).

bioRxiv preprint doi: https://doi.org/10.64898/2026.03.03.709366; this version posted March 3, 2026. The copyright holder for this preprint (which was not certified by peer review) is the author/funder, who has granted bioRxiv a license to display the preprint in perpetuity. It is made available under aCC-BY 4.0 International license.

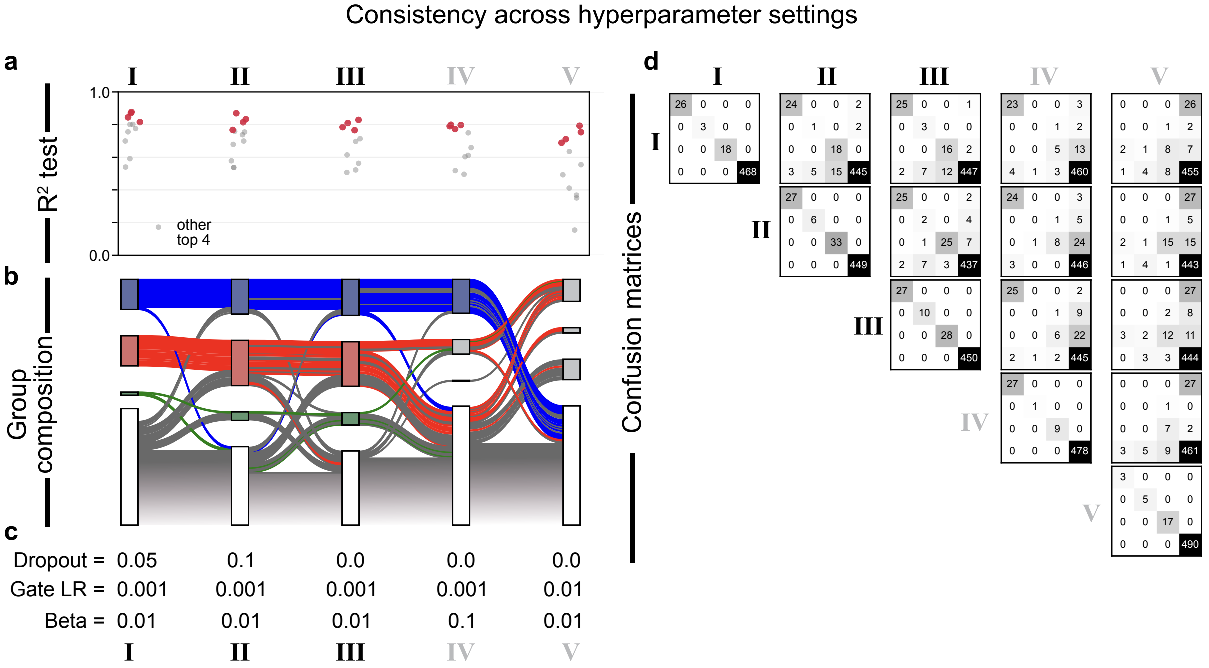

Supplementary Figure S13: Consistency of groups across hyperparameters: Tara metagenomics. Here we evaluate the sensitivity of the learned gene groupings from Fig. 4 to training hyperparameters. (a) R^2 values for each of the different hyperparameter sets, enumerated with Roman numerals. Small dots correspond to single models in the ensemble. The top four models highlighted in red are used to determine the consensus grouping (Methods). Performance is mostly constant for hyperparameter sets I-III but begins to degrade in IV and V. (b) Consensus group composition resulting for each hyperparameter set. Each set of nodes (rectangles) in the flow diagram are aligned vertically with the corresponding hyperparameter set from (a). Each bar between nodes represents one gene module; its thickness corresponds to its average abundance. The genes in each of the three groups from Fig. 4 are colored accordingly. The bottom group (gray) represents all genes that are removed by the gating procedure. These are not used as an input to the structure-function map neural network. (c) The hyperparameters corresponding to each set I-V. (d) Confusion matrices to compare the group assignments across hyperparameter sets. Each matrix is a comparison between one set of hyperparameters (Roman numerals). Entry i, j in the matrix is the number of genes that are assigned to group i for one hyperparameter set and to group j for another hyperparameter set.

bioRxiv preprint doi: https://doi.org/10.64898/2026.03.03.709366; this version posted March 3, 2026. The copyright holder for this preprint (which was not certified by peer review) is the author/funder, who has granted bioRxiv a license to display the preprint in perpetuity. It is made available under aCC-BY 4.0 International license.

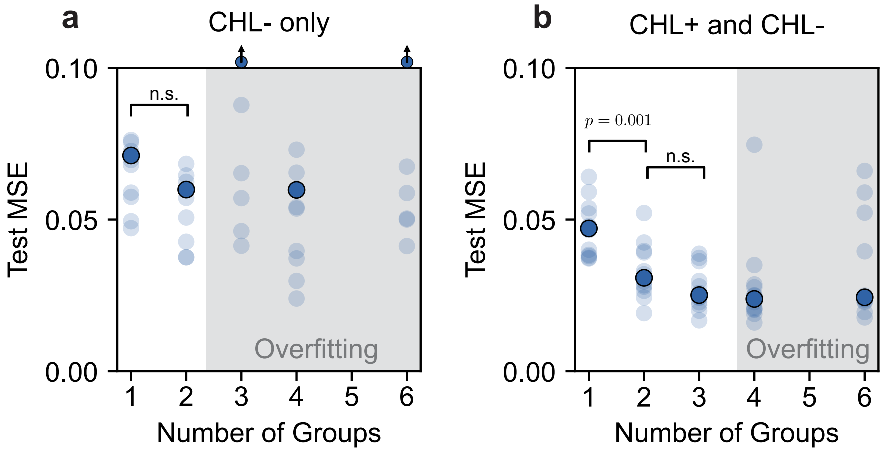

Supplementary Figure S14: Test (mean squared) error versus number of groups for soil data. Predictions are made with ASV-level abundance data as input, using only CHL-untreated samples (a) or both treated and untreated samples (b). Models are trained with the gating procedure described in Methods. Each semi-transparent point is one model trained with a different test-train split of the data. Solid point is the median loss. There are clear signs of overfitting for larger numbers of groups (gray background) because test error begins to grow rapidly. Note that some models with test MSE > 0.10 are not shown. Cases where the median MSE is greater than this value are shown with a point lying on top of the axis in (a). Parameters $lr = 10^{-3}$, $lr_{\text{gate}} = 10^{-1}$, $\beta = 10^{-2}$, $p_{\text{dropout}} = 0.05$. p-values shown for two-sided t-test comparing the means of the distribution of errors. Other differences are not significant (n.s.); for CHL- only the differences between 1 and 2 groups has $p = 0.28$ and for both treated and non-treated the difference between 2 and 3 groups is $p = 0.10$.

bioRxiv preprint doi: https://doi.org/10.64898/2026.03.03.709366; this version posted March 3, 2026. The copyright holder for this preprint (which was not certified by peer review) is the author/funder, who has granted bioRxiv a license to display the preprint in perpetuity. It is made available under aCC-BY 4.0 International license.

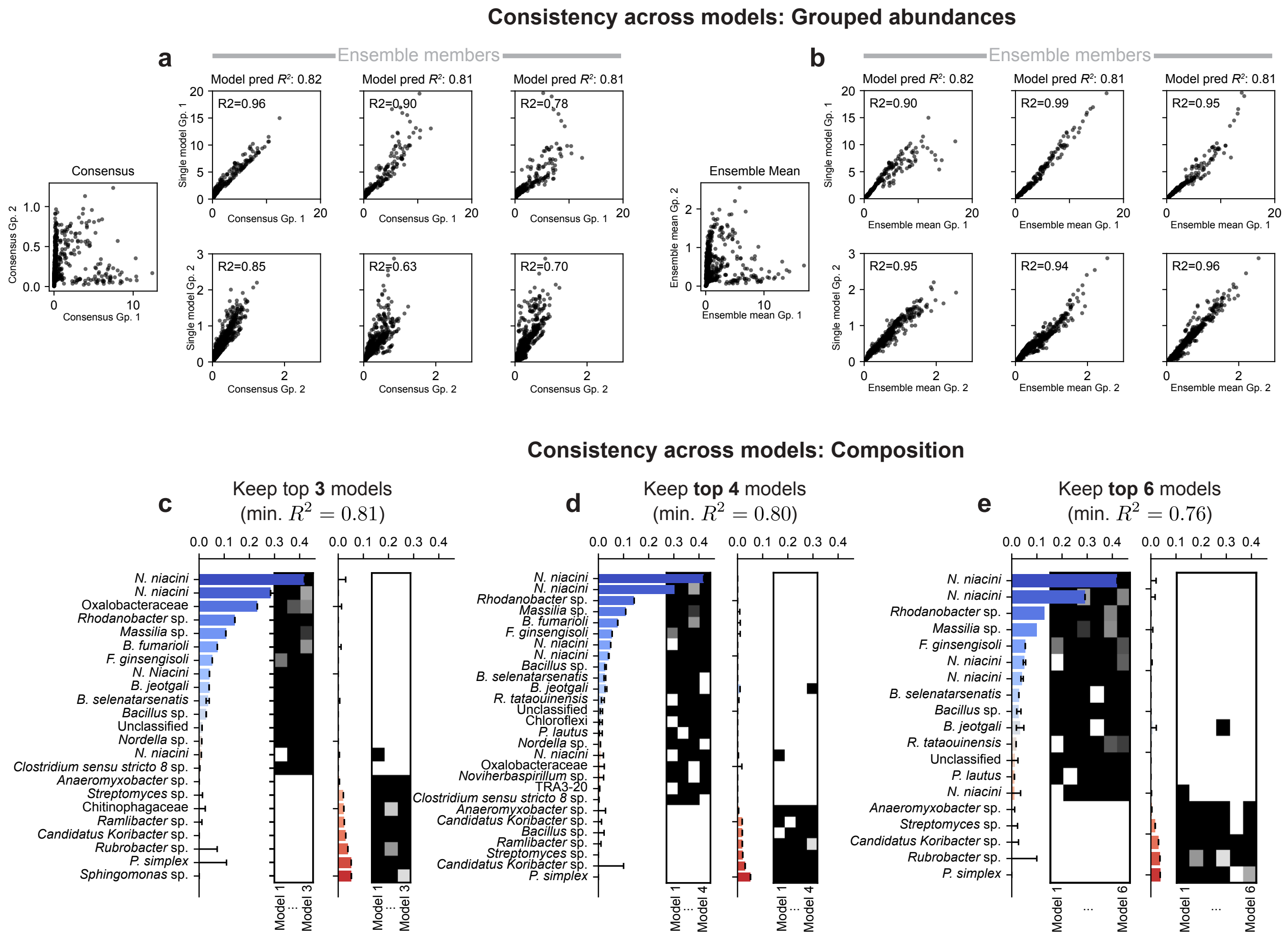

Supplementary Figure S15: Learned assignments are consistent across different selection criteria. We train an ensemble of models, each of which may learn a different grouping. We explore two ways of determining "average" group abundances. First, we can take a "consensus grouping" which selects all species that are placed into a single group more than 50% of the time across the ensemble (see Methods). Second, we can first compute the group abundances for each ensemble member individually, and then compute the average across the ensemble. These give different results because in the first approach one neglects small or unimportant groups, while in the second they are taken into account because they contribute to the group abundance for each individual model. (a) Comparison of group 1 and 2 abundances (top and bottom row) for the individual models compared to the consensus group. (b) Comparison of individual model group abundances to the ensemble mean abundances. (c-d) Show how our selection of the consensus group members is stable whether we take the top 25% (3) of models or the top 50% (6) to build the consensus group. From this subset of models, a group member is included if it is present in 75% or more of the individual models. (c) Left axis shows the average end-point abundances (blue) of each species included in the consensus group. The inset matrix shows the presence of each species across all models. Right axis shows abundances (red) of species in Group 2. These species are all those included if we determine our consensus group from the top 3 performing models. (d) Consensus group members derived from the top 4 performing models, sorted by abundance (blue, red) with their presence/absence across models (inset matrices). (e) Consensus group members derived from the top 6 performing models.

bioRxiv preprint doi: https://doi.org/10.64898/2026.03.03.709366; this version posted March 3, 2026. The copyright holder for this preprint (which was not certified by peer review) is the author/funder, who has granted bioRxiv a license to display the preprint in perpetuity. It is made available under aCC-BY 4.0 International license.

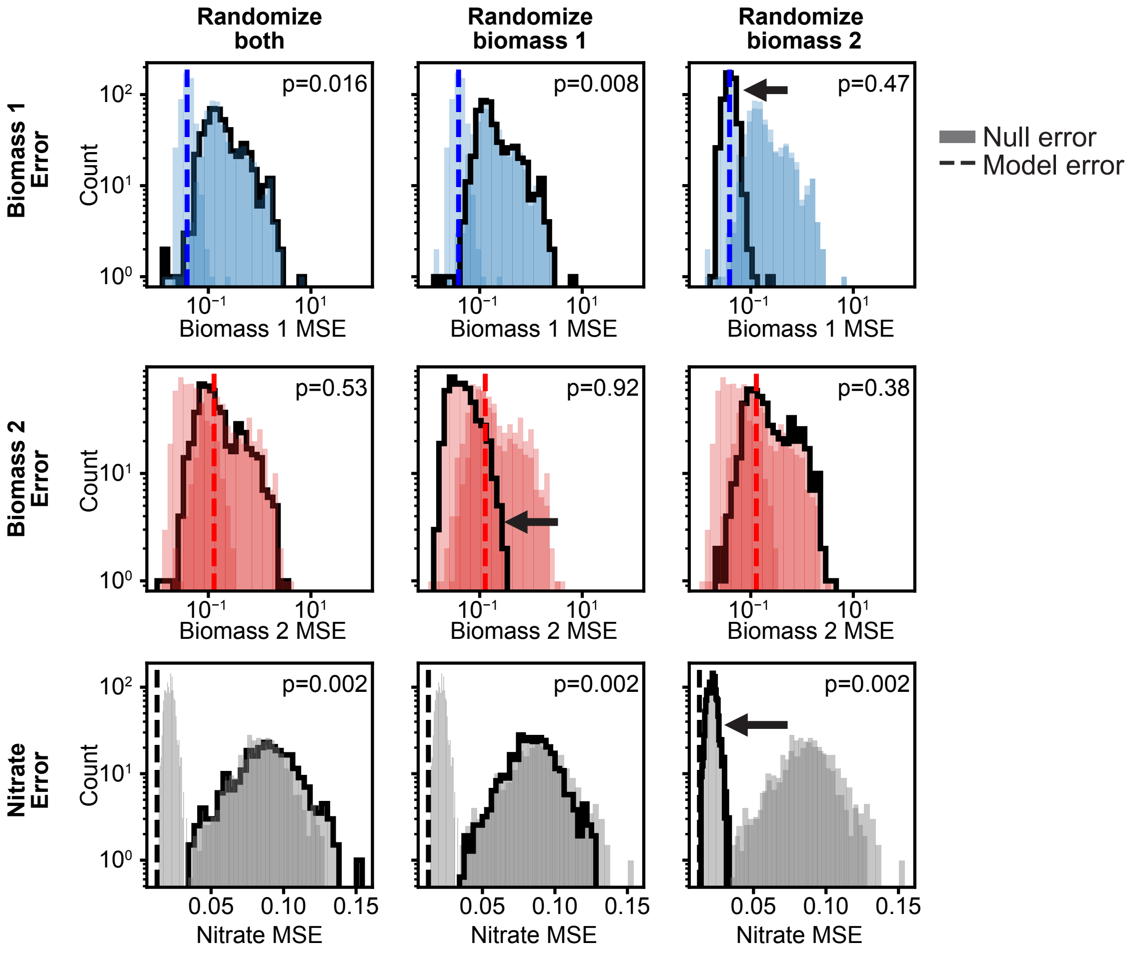

Supplementary Figure S16: Null model comparison. A full description of the figure is found in Methods. Each row shows the distribution (highlighted in black) of errors using a null model of group abundances which draws either both groups from a null distribution (left column), only group 1 (middle) or only group 2 (right). Distributions from other null models are shown, not highlighted, for comparison. Top row shows error in biomass 1 end-point abundance prediction; middle shows error for biomass 2; bottom shows error for nitrate dynamics. p-values shown are the empirical probabilities of observing a null model error that is lower than the correct model error (shown with vertical dashed line). The arrows highlight some observations which are discussed in Methods.

bioRxiv preprint doi: https://doi.org/10.64898/2026.03.03.709366; this version posted March 3, 2026. The copyright holder for this preprint (which was not certified by peer review) is the author/funder, who has granted bioRxiv a license to display the preprint in perpetuity. It is made available under aCC-BY 4.0 International license.

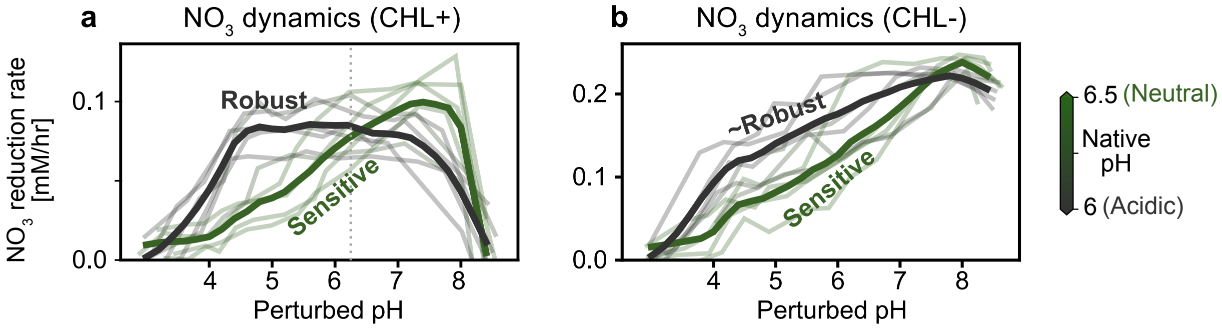

Supplementary Figure S17: Nitrate reduction dynamics in CHL treated and untreated conditions. (a) Reproduction of Fig. 6c for comparison. (b) Nitrate reduction rate for each soil sample as a function of perturbed pH. As in Fig. 6c, each soil sample (faint lines) is colored by native pH. Solid lines show the average across acidic (pH < 6.25) and neutral (pH > 6.25) soils. The difference between robust and sensitive soils is less pronounced than in the CHL-treated samples but still present.
